# Galactomannan Is a Biomarker of Fosmanogepix (APX001) Efficacy in Treating Experimental Invasive Pulmonary Aspergillosis

**DOI:** 10.1128/AAC.01966-19

**Published:** 2019-12-20

**Authors:** Teclegiorgis Gebremariam, Sondus Alkhazraji, Yiyou Gu, Shakti Singh, Abdullah Alqarihi, Karen Joy Shaw, Ashraf S. Ibrahim

**Affiliations:** a The Lundquist Institute for Biomedical Innovations, Torrance, California, USA; b Amplyx Pharmaceuticals Inc., San Diego, California, USA; c David Geffen School of Medicine, University of California at Los Angeles, Los Angeles, California, USA

**Keywords:** APX001, APX001A, Gwt1, antifungal, *Aspergillus*, infection model, 1-aminobenzotriazole, manogepix, fosmanogepix, galactomannan, *Aspergillus fumigatus*, antifungal agents, murine

## Abstract

Galactomannan (GM) detection in biological samples has been shown to predict therapeutic response by azoles and polyenes. In a murine invasive pulmonary aspergillosis model, fosmanogepix or posaconazole treatment resulted in an ∼6- to 7-log reduction in conidial equivalents (CE)/g lung tissue after 96 h versus placebo. Changes in GM levels in BAL fluid and serum mirrored reductions in lung CE, with significant decreases seen after 96 h or 72 h for fosmanogepix or posaconazole, respectively (*P* < 0.02).

## Abstract (precis)

Galactomannan (GM) detection in biological samples has been shown to predict therapeutic response by azoles and polyenes. In a murine invasive pulmonary aspergillosis model, fosmanogepix or posaconazole treatment resulted in an ∼6- to 7-log reduction in conidial equivalents (CE)/g lung tissue after 96 h versus placebo.

## TEXT

Invasive pulmonary aspergillosis (IPA) is a serious fungal infection affecting immunocompromised patients. Fosmanogepix (APX001) is a first-in-class antifungal prodrug that is currently in clinical development for the treatment of invasive fungal infections (1, 2). Fosmanogepix is rapidly and completely metabolized by systemic phosphatases to the active moiety manogepix (MGX; previously APX001A), which targets the highly conserved fungal enzyme Gwt1. Gwt1 catalyzes an early step in glycosylphosphatidylinositol (GPI) anchor biosynthesis (3, 4), and inhibition of this enzyme has pleiotropic effects on cell wall integrity, biofilm formation, and fungal growth (5, 6).

We previously reported the efficacy of fosmanogepix in treating murine IPA in immunocompromised mice (7). In that study, we administered 50 mg/kg of body weight of the cytochrome P450 inhibitor 1-aminobenzotriazole (ABT) 2 h prior to fosmanogepix to extend the short half-life of MGX in mice (from a range of 1.4 to 2.75 h to 9 h). The ABT treatment also enhanced MGX exposures (area under the concentration-time curve [AUC]), more closely mimicking phase 1 clinical exposures where a long half-life was observed (half-life [*t*_1/2_], 2 to 2.5 days in humans) (1, 2, 7). The extended half-life of MGX (administered as 78 mg/kg once a day [QD] or 104 mg/kg QD of fosmanogepix) significantly enhanced mouse survival and reduced lung fungal burden by several log conidial equivalents (CE)/gram tissue versus the untreated control. Importantly, we determined that ABT has no *in vitro* antimicrobial activity against Aspergillus fumigatus at the administered dose of 50 mg/kg, which results in a *C*_max_ of 17 μg/ml in rats. Furthermore, when higher ABT concentrations were evaluated (up to 250 μg/ml), no antifungal activity was observed (7). Concordant with the lack of *in vitro* activity, ABT treatment alone did not enhance survival time nor reduce tissue fungal burden compared with infected mice without treatment in several animal models, including IPA (7), cryptococcal meningitis (8), or Coccidioides immitis pneumonia (9). Therefore, the survival benefit seen in mice receiving fosmanogepix+ABT is solely due to the effect of MGX.

Galactomannan (GM) detection in biological samples using the Platelia enzyme-linked immunosorbent assay (ELISA) has been shown to predict therapy response by azoles and polyenes (10). Here, we investigated the potential use of GM as a biomarker of fosmanogepix efficacy in the immunosuppressed murine model of IPA.

## 

### Effects of fosmanogepix and posaconazole in a murine model of IPA.

Male ICR mice were rendered neutropenic by administration of 200 mg/kg cyclophosphamide and 500 mg/kg cortisone acetate on day −2 and day +3, relative to infection. Mice were infected (day 0) with A. fumigatus AF293 via inhalation (11) and treated orally 16 h later with fosmanogepix (78 mg/kg QD or 104 mg/kg QD) or posaconazole (20 mg/kg QD or 30 mg/kg BID, a dose which achieves exposures in mice equivalent to 6 times the clinical exposures in humans [12]). To prevent bacterial infection, 50 μg/ml enrofloxacin (Bayer, Leverkusen, Germany) was added to the drinking water from day −3 to day 0. Ceftazidime (5 μg/dose/0.2 ml) replaced enrofloxacin treatment on day 0 and was administered daily by subcutaneous injection from day 0 until time of sacrifice. As in our previous study, mice were administered 50 mg/kg of the ABT 2 h prior to fosmanogepix or placebo treatment to extend the MGX half-life and increase MGX exposure (7). Treatments continued until sacrifice at 48, 72, and 96 h postinfection, at which time the lungs, bronchoalveolar lavage (BAL) fluid, and sera were collected. Lung fungal burden was assessed by CE using qPCR (7, 13), while GM was determined using the Platelia ELISA kit (Bio-Rad). The GM index was calculated as the optical density (OD) value of the specimen divided by the mean OD of the wells containing a cutoff control provided in the kit. Values of an index of <0.50 and >0.50 are considered negative and positive for GM, respectively.

### Effects on log_10_ CE/g lung tissue.

**FIG 1:**
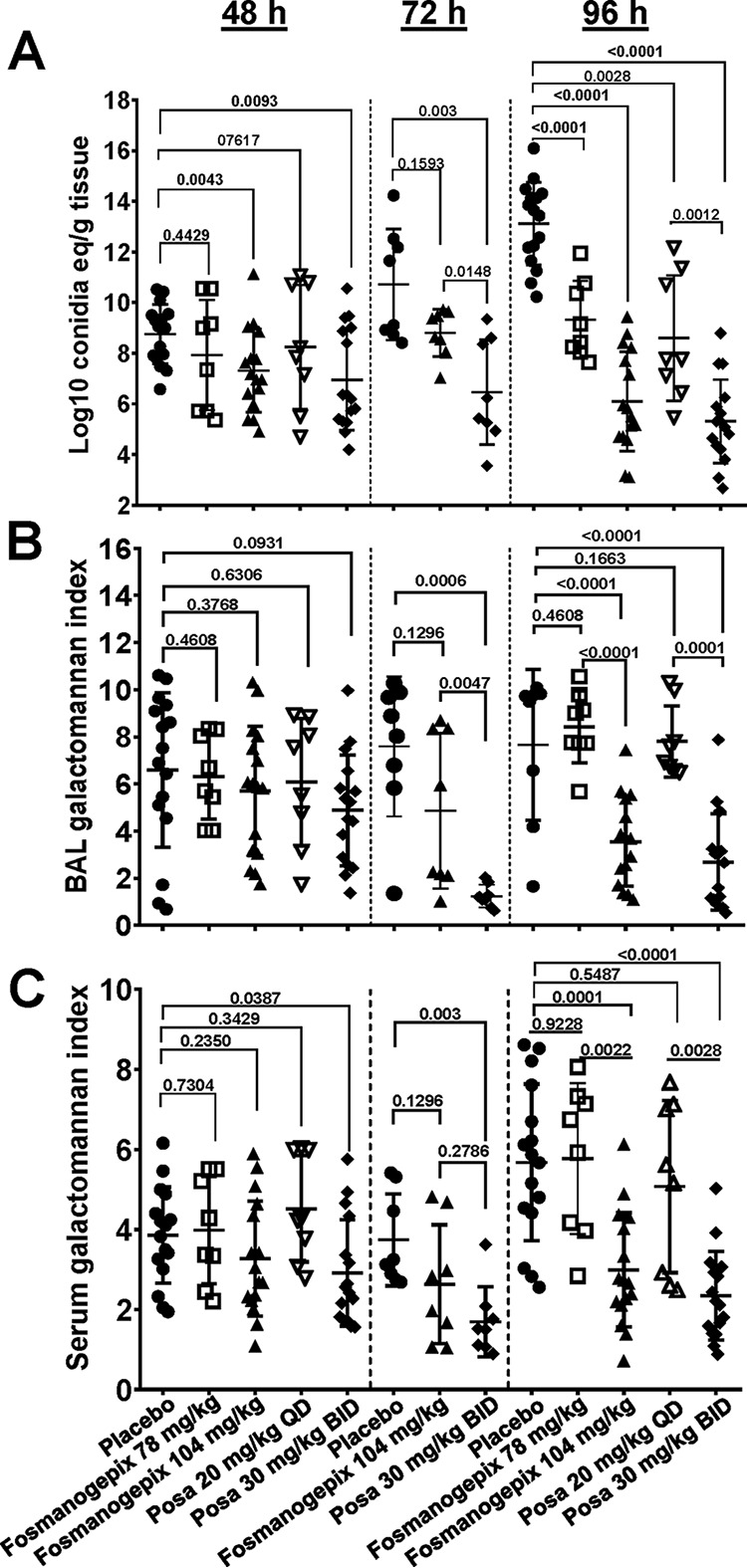
Effect of antifungal treatment on log_10_ CE/g lung tissue (A), BAL fluid GM (B), and serum GM (C) levels. Mice (*n* = 8 to 16 mice/group) were infected with A. fumigatus (average inhaled inoculum of 5.1 × 10^3^ conidia from 2 experiments). Data were presented as medians ± interquartile ranges) and evaluated using the nonparametric Wilcoxon signed-rank test (Prism 5; GraphPad Software, Inc., San Diego, CA). The *y* axis 2.0 value in (A) represents the lower limit of detection of the assay. Posa, posaconazole.

The data in Fig. 1A show a time- and dose-dependent reduction in log_10_ CE for fosmanogepix and posaconazole. At 48 h, only the suprahumanized dose of posaconazole (30 mg/kg twice a day [BID], which achieves exposures in mice that are 6 times higher than exposures in humans [12]) and the higher dose of fosmanogepix (104 mg/kg) showed a significant reduction in log_10_ CE/g lung tissue versus placebo control (*P* = 0.009 and *P* = 0.004, respectively). At 72 h, CE/g lung tissue for the placebo increased by ∼2 logs versus the 48 h placebo. The 30 mg/kg BID dose of posaconazole showed a significant reduction in CE versus placebo (*P* = 0.003), whereas the higher dose of fosmanogepix did not. This may be due to the lower number of animals used in the 72 h cohort (*n* = 8 per group). At 96 h, CE for the placebo increased by over 4 logs versus the 48-h placebo. At 96 h, all treatment groups demonstrated a reduction in fungal burden compared with the 96-h placebo (*P* ≤ 0.002). Dose-dependent reductions were observed for both fosmanogepix and posaconazole, with the higher dose of posaconazole (30 mg/kg BID) being equivalent to the higher dose of fosmanogepix (104 mg/kg; *P* = 0.25). Similarly, the lower dose of posaconazole (20 mg/kg QD) was equally efficacious as 78 mg/kg of fosmanogepix in reducing log_10_ CE/g of lung (*P* = 0.37). Thus, although the suprahumanized dose of posaconazole resulted in a faster reduction in lung fungal burden after 72 h, both drugs resulted in similar reductions (∼6 to 7 log) in lung CE versus placebo after 96 h.

### Effects on GM levels in BAL fluid.

Changes in BAL fluid GM index levels are shown in Fig. 1B. At 48 h, all treatment cohorts demonstrated GM levels that ranged from 4.9 to 6.5 and were statistically equivalent (*P* ≥ 0.09) to the infected placebo group with a GM index of 7.2. At 72 h, 30 mg/kg BID posaconazole demonstrated a significant reduction in GM versus both placebo (reduction in GM index of 7.3; *P* = 0.0006) and 104 mg/kg fosmanogepix (reduction in GM index of 4.4; *P* = 0.0047). Fosmanogepix GM was equivalent to placebo (*P* = 0.13). At 96 h, a significant reduction in the GM index was observed only for the high dose of posaconazole and fosmanogepix (*P* < 0.0001). These data are consistent with the observed reductions in log_10_ CE/g lung tissue (Fig. 1A); however, there may be a delay in the reduction in the BAL fluid GM index versus CE.

### Effects on GM levels in serum.

Similar to what was observed for BAL fluid GM, only the 30-mg/kg BID high dose of posaconazole resulted in a significant decrease in the serum GM index versus placebo at both 48 h (*P* = 0.0385) and 72 h (*P* = 0.003). Likewise, at 96 h, a significant reduction in the GM index in serum was observed for both the high dose of posaconazole and fosmanogepix (*P* ≤ 0.0001). Thus, both serum and BAL fluid GM index reductions were related in terms of the dose of both antifungals and time required.

Changes in GM indexes in BAL fluid or serum samples mirrored reductions in lung CE, with significant decreases seen after 96 h or 72 h for fosmanogepix or posaconazole, respectively, versus placebo (*P* < 0.02) at doses that reflect a clinically relevant dose of fosmanogepix (1, 2) and a suprahumanized dose of posaconazole (12). At 96 h, fosmanogepix was as effective as posaconazole in reducing fungal burden and GM from BAL fluid and serum collected from immunosuppressed mice with IPA. These time- and dose-dependent reductions of lung fungal burden and GM levels suggest that GM can be used as a biomarker of fosmanogepix efficacy in immunosuppressed mice. Continued investigation of fosmanogepix as a novel antifungal agent against aspergillosis is warranted.
